# AR12 increases BAG3 expression which is essential for Tau and APP degradation via LC3-associated phagocytosis and macroautophagy

**DOI:** 10.18632/aging.204337

**Published:** 2022-10-13

**Authors:** Paul Dent, Laurence Booth, Jane L. Roberts, Andrew Poklepovic, Jennifer Martinez, Derek Cridebring, Eric M. Reiman

**Affiliations:** 1 Department of Biochemistry and Molecular Biology, Virginia Commonwealth University, Richmond, VA 23298, USA; 2 Department of Surgery, Virginia Commonwealth University, Richmond, VA 23298, USA; 3 Department of Medicine, Virginia Commonwealth University, Richmond, VA 23298, USA; 4 National Institute of Environmental Health Sciences, Inflammation and Autoimmunity Group, Triangle Park, Durham, NC 27709, USA; 5 Translational Genomics Research Institute (TGen), Phoenix, AZ 85004, USA; 6 Banner Alzheimer’s Institute, Phoenix, AZ 85006, USA

**Keywords:** Alzheimer’s disease, macroautophagy, LAP / LANDO, GRP78, AR12

## Abstract

We defined the mechanisms by which the chaperone ATPase inhibitor AR12 and the multi-kinase inhibitor neratinib interacted to reduce expression of Tau and amyloid-precursor protein (APP) in microglia and neuronal cells. AR12 and neratinib interacted to increase the phosphorylation of eIF2A S51 and the expression of BAG3, Beclin1 and ATG5, and in parallel, enhanced autophagosome formation and autophagic flux. Knock down of BAG3, Beclin1 or ATG5 abolished autophagosome formation and significantly reduced degradation of p62, LAMP2, Tau, APP, and GRP78 (total and plasma membrane). Knock down of Rubicon, a key component of LC3-associated phagocytosis (LAP), significantly reduced autophagosome formation but not autophagic flux and prevented degradation of Tau, APP, and cell surface GRP78, but not ER-localized GRP78. Knock down of Beclin1, ATG5 or Rubicon or over-expression of GRP78 prevented the significant increase in eIF2A phosphorylation. Knock down of eIF2A prevented the increase in BAG3 expression and significantly reduced autophagosome formation, autophagic flux, and it prevented Tau and APP degradation. We conclude that AR12 has the potential to reduce Tau and APP levels in neurons and microglia via the actions of LAP, endoplasmic reticulum stress signaling and macroautophagy. We hypothesize that the initial inactivation of GRP78 catalytic function by AR12 facilitates an initial increase in eIF2A phosphorylation which in turn is essential for greater levels of eIF2A phosphorylation, greater levels of BAG3 and macroautophagy and eventually leading to significant amounts of APP/Tau degradation.

## INTRODUCTION

AR12 (OSU-03012) is a derivative of the anti-inflammatory agent celecoxib; unlike the parent compound AR12 lacks COX2 inhibitory activity. Our group demonstrated that AR12 reduced the expression of many chaperone proteins and caused endoplasmic reticulum stress signaling with repression of protein translation, i.e., increased eIF2α S51 phosphorylation, and rapidly caused autophagosome formation, followed later by autolysosome formation, i.e., autophagic flux [1–10]. We demonstrated that the key cellular target for AR12 was the ER stress-regulatory chaperone GRP78 (aka BiP, HSPA5), followed by other ATP-dependent chaperones in the HSP90/HSP70 families. AR12 was shown to directly inhibit chaperone ATPase activities. *In vivo* studies using AR12 in mice, rats and rabbits has shown drug efficacy without damage to normal tissues [8, 11].

In our recent studies in Alzheimer’s Disease, using wild type and genetically modified HCT116 colon cancer cells as a model system expressing either ATG16L1 T300 or ATG16L1 A300, we determined whether drugs that directly inhibit the chaperone ATPase activity or cause chaperone degradation and endoplasmic reticulum stress signaling leading to macroautophagy could reduce the levels of proteins which play a pathogenic role in neurodegenerative diseases [10]. AR12 and the breast cancer drug neratinib (NER) rapidly reduced expression of Tau, amyloid precursor protein (APP), superoxide dismutase 1 (SOD1) and TAR DNA-binding protein 43 (TDP-43) [10, 12–14].

GRP78 is expressed in the ER and on the outer leaflet of the plasma membrane [15–20]. In the ER it prevents ER stress signaling by PERK, IRE1 and ATF6 and acts as a molecular chaperone renaturing misfolded proteins. GRP78 can chaperone Tau and APP, and can prevent, in an ATP-independent fashion, the processing of APP to insoluble amyloid-β [21]. Cell surface GRP78 plays a key role in permitting neurons and microglia to uptake Tau and APP, i.e., GRP78 is a key player in facilitating the ‘prion’-like bystander behavior of Tau [21]. In that regard, GRP78 has already been shown to modulate prion propagation [19]. At first glance, approaches that would enhance GRP78 expression, rather than inhibiting its ATPase, could be considered as an AD therapeutic approach. This would prevent protein denaturation and processing of Tau and APP into tangles. However, over-expression of GRP78 also blocks both ER stress signaling and the ability of cells to perform macroautophagy and autophagic flux. i.e., over-expression of GRP78 can stabilize Tau and APP, but it does so at the cost of preventing their degradation. Over-expression of GRP78 will also increase the amount of Tau and APP being taken up by bystander neurons and microglia.

We know that AR12 and neratinib have overlapping and separate biologies which facilitate autophagosome formation and autophagic flux resulting in the degradation of proteins. We hypothesize that AR12, by inhibiting GRP78, leads to increased ER stress signaling and a reduced capacity to signal into the PI3K pathway, resulting in the inactivation of mTOR, with subsequent autophagosome formation and degradation of Tau and APP. Neratinib through reactive oxygen species, activates ataxia telangiectasia mutated (ATM) which phosphorylates and activates the AMP-dependent protein kinase (AMPK). AMPK, by acting to reduce mTOR activity and by directly activating ULK1, also results in autophagosome formation and degradation of Tau and APP. Furthermore, because neratinib can cause degradation of HER2, which is over-expressed in the brains of AD patients, PI3K/mTOR signaling will be further reduced [12–14, 22, 23]. The present studies were performed to define the biology of AR12 and neratinib in macrophages, microglia, and neuronal cells and whether AR12, alone or combined with neratinib, was competent to cause degradation of APP and Tau proteins in these cell types.

## MATERIALS AND METHODS

### Materials

HCT116 colon cancer cells and HCN2 neuronal cells were purchased from the ATCC (Bethesda, MD). BV2 rodent microglial cells and RAW macrophages were supplied by Dr. Martinez. AR12 was purchased from Selleckchem (Houston, TX). Neratinib was supplied by Puma Biotechnology Inc. (Los Angeles, CA). Plasmids to express HSP70, HSP90, LC3-GFP-RFP, Tau-GFP, and amyloid precursor protein (APP)-FLAG were purchased from Addgene (Watertown, MA). The plasmid to express GRP78 was kindly provided by Dr. Amy Lee (University of Southern California, Los Angeles). Trypsin-EDTA, RPMI, penicillin-streptomycin were purchased from GIBCOBRL (GIBCOBRL Life Technologies, Grand Island, NY). Other reagents and performance of experimental procedures were as described [1–14]. Cell Signalling antibodies: ATM (D2E2) Rabbit mAb #2873; Phospho-ATM (Ser1981) (D25E5) Rabbit mAb #13050; AMPKα #2532; Phospho-AMPKα (Thr172) (D4D6D) Rabbit mAb #50081; mTOR #2972; Phospho-mTOR (Ser2448) #2971; Phospho-mTOR (Ser2481) #2974; ULK1 (R600) #4773; Phospho-ULK1 (Ser317) #37762; Phospho-ULK1 (Ser757) #6888; eIF2α #9722; Phospho-eIF2α (Ser51) #9721; PERK (D11A8) Rabbit mAb #5683; Phospho-PERK (Thr980) (16F8) Rabbit mAb #3179; AKT Antibody #9172; Phospho-AKT (Thr308) (244F9) Rabbit mAb #4056; STAT3 (124H6) Mouse mAb #9139; Phospho-STAT3 (Tyr705) Antibody #9131; STAT5 (D2O6Y) Rabbit mAb #94205; Phospho-STAT5 (Tyr694) #9351; Beclin-1 #3738; ATG5 (D5F5U) Rabbit mAb #12994; ATG13 (D4P1K) Rabbit mAb #13273; Phospho-ATG13 (Ser355) (E4D3T) Rabbit mAb #46329; GRP78/BiP #3183; CHOP (L63F7) Mouse mAb #2895 PP1α Antibody #2582; NFκB p65 (L8F6) Mouse mAb #6956; Phospho-NFκB p65 (Ser536) (93H1) Rabbit mAb #3033; Src (36D10) Rabbit mAb #2109; Phospho-Src Family (Tyr416) (E6G4R) Rabbit mAb #59548; Phospho-Src (Tyr527) Antibody #2015; c-MET (25H2) Mouse mAb # 3127; Phospho-MET (Tyr1234/1235) Antibody #3126; FAS (4C3) Mouse mAb #8023; FAS-L (D1N5E) Rabbit mAb #68405; JAK1/2 (6G4) Rabbit mAb #3344; Phospho-Jak1 (Tyr1034/1035)/Jak2 (Tyr1007/1008) (E9Y7V) Mouse mAb #66245; c-KIT (D13A2) XP® Rabbit mAb #3074; Phospho-c-KIT (Tyr719) Antibody #3391; HER/ErbB Family Antibody Sampler Kit #8339; p70 S6 Kinase #9202; Phospho-p70 S6 Kinase (Thr389) #2904; PDGF Receptor beta #3164; Phospho-PDGF Receptor beta (Tyr754) (23B2) Rabbit mAb #2992; Phospho-p44/42 MAPK (Erk1/2) (Thr202/Tyr204) (20G11) Rabbit mAb #4376; Histone Deacetylase (HDAC) Antibody Sampler Kit #9928; HDAC7 (D4E1L) Rabbit mAb #33418; HDAC8 (E7F5K) Rabbit mAb #66042; HDAC11 (D5I8E) Rabbit mAb #58442; MHC Class II (LGII-612.14) Mouse mAb #68258; p38 MAPK #9212; Phospho-p38 MAPK (Thr180/Tyr182) (3D7) Rabbit mAb #9215; LATS1 (C66B5) Rabbit mAb #3477; Phospho-LATS1/2 (Ser909) #9157; Phospho-LATS1/2 (Thr1079) (D57D3) Rabbit mAb #8654; YAP (1A12) Mouse mAb #12395; Phospho-YAP (Ser127) (D9W2I) Rabbit mAb #13008; Phospho-YAP (Ser109) (E5I9G) Rabbit mAb #53749; Phospho-YAP (Ser397) (D1E7Y) Rabbit mAb #13619; TAZ (E8E9G) Rabbit mAb #83669 Phospho-TAZ (Ser89) (E1X9C) Rabbit mAb #59971; NEDD4 Antibody #2740; PTEN Antibody #9552; Estrogen Receptor α (D6R2W) Rabbit mAb #13258; Cyclin Antibody Sampler Kit #9869; BCL-XL #2762; MCL-1 (D35A5) Rabbit mAb #5453; BAX #2772; BAK #2814; BIM #2819; JNK1/2 #9252; Phospho-JNK (Thr183/Tyr185) (81E11) Rabbit mAb #4668; p44/42 MAPK (ERK1/2) (L34F12) Mouse mAb #4696). Santa Cruz Biotechnology antibodies: Histone Deacetylase 9 (HDAC9) (B-1) #sc398003; Histone Deacetylase 10 (HDAC10) (E-2) #393417. ABCAM antibodies: Anti-PD-L1 [28-8] (ab205921); Anti-PD-L2 [EPR25200-50] (ab288298); Anti-Ornithine Decarboxylase/ODC [ODC1 / 2878R] (ab270268); BAG3 ab92309; HSP90 (#2928); HSP90 (ab195575); HSP90 3G3 (13495); GRP78 (ab191023); GRP78 (ab103336); HSP27 [EP1724Y] (ab62339).

**Figure 1:**
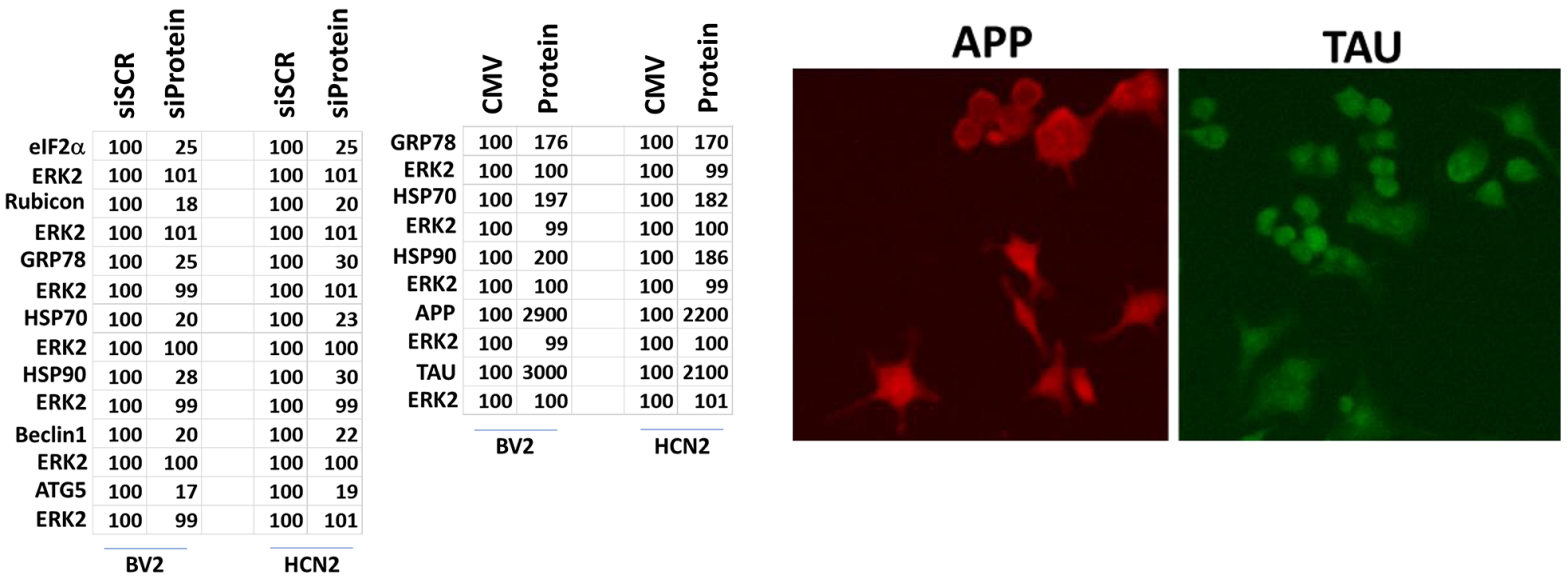
**Control data showing protein expression knock down and protein over-expression.** Left: BV2 and HCN2 cells as indicated were transfected with siRNA molecules to knock down the expression of the indicated proteins or transfected with plasmids to over-express the indicated proteins. The percentage remaining after knock-down or the percentage over-expression above basal levels is indicated. (n = 3 +/-SD) (total ERK2 is included as an invariant total protein loading control). Right: Images of HCN2 cells transfected to express TAU or APP.

Specific multiple independent siRNAs to knock down expression were purchased from Qiagen (Hilden, Germany). Human**:** HSP90 GeneGlobe ID SI03028606; HSP70 GeneGlobe ID SI04324481; GRP78 GeneGlobe ID SI00443114; Beclin-1 GeneGlobe ID SI00055573; ATG5 GeneGlobe ID SI00069251; Rubicon GeneGlobe ID SI00452592; BAG3 GeneGlobe ID SI02632812; AMPKα GeneGlobe ID SI00086387; eIF2α GeneGlobe ID SI00105784; ULK1 GeneGlobe ID SI00053060; perk GeneGlobe ID SI00069048. Mouse**:** Beclin-1 GeneGlobe ID SI00214165; ATG5 GeneGlobe ID SI00230664; BAG3 GeneGlobe ID SI00208425; AMPKα GeneGlobe ID SI01388247; eIF2α GeneGlobe ID SI00969675; ULK1 GeneGlobe ID SI01461999; PERK GeneGlobe ID SI00991319. Thermo Fisher mouse: HSP70 si RNA ID: s201487 Cat #4390771; GRP78 si RNA ID: s67084 Cat #4390771; Rubicon si RNA ID: s104761 Cat #4390771; HSP90 si RNA ID: s67897 Cat #4390771. Multiple control studies have been previously presented showing on-target specificity of our siRNAs, primary antibodies, and our phospho-specific antibodies to detect both total protein levels and phosphorylated levels of proteins and we present data in HCN2 (human) and BV2 (mouse) cells (Figure 1) [1–14].

### Methods

Cells were grown at 37° C (5% (v/v CO_2_) using RPMI supplemented 5% (v/v) fetal calf serum and 1% (v/v) Non-essential amino acids. All therapeutics were dissolved in DMSO making a 10 mM stock solution, stored in multiple 100 μl vials. AR12 and neratinib are diluted in DMSO until the final dilution into growth media (VEH: vehicle control; NER: neratinib). The final concentration of DMSO is never more than 0.1% (v/v). Cells were not cultured in reduced serum media.

### Assessments of protein expression and protein phosphorylation

Multi-channel fluorescence HCS microscopes perform true in-cell western blotting. Three independent cultures derived from three thawed vials of cells of a tumor were sub-cultured into individual 96-well plates (~5,000 cells per well). Twenty-four hours after plating, the cells are transfected with a control plasmid or a control siRNA, or with an empty vector plasmid or with plasmids to express various proteins. After another 24 hours, the cells are ready for drug exposure(s). At various time-points after the initiation of drug exposure, cells are fixed in place using paraformaldehyde and using Triton X100 for permeabilization. Standard immunofluorescent blocking procedures are employed, followed by incubation of different wells with a variety of validated primary antibodies and subsequently validated fluorescent-tagged secondary antibodies are added to each well. The microscope determines the background fluorescence in the well and in parallel randomly determines the mean fluorescent intensity of 100 cells per well. The counting is independent of cell density. Of note for scientific rigor is that the operator does not personally manipulate the microscope to examine specific cells; the entire fluorescent accrual method is independent of the operator.

### Transfection of cells with siRNA or with plasmids

### For plasmids

Cells were plated and 24h after plating, transfected. Plasmids expressing a specific mRNA or appropriate empty vector control plasmid (CMV) DNA was diluted in 50 μl serum-free and antibiotic-free medium (1 portion for each sample). Concurrently, 2 μl Lipofectamine 2000 (Invitrogen), was diluted into 50 μl of serum-free and antibiotic-free medium (1 portion for each sample). Diluted DNA was added to the diluted Lipofectamine 2000 for each sample and incubated at room temperature for 30 min. This mixture was added to each well / dish of cells containing 100 μl serum-free and antibiotic-free medium for a total volume of 300 μl, and the cells were incubated for 4 h at 37° C. An equal volume of 2x serum containing medium was then added to each well. Cells were incubated for 24h, then treated with drugs.

### Transfection for siRNA

Cells from a fresh culture growing in log phase as described above, and 24h after plating transfected. Prior to transfection, the medium was aspirated, and serum-free medium was added to each plate. For transfection, 10 nM of the annealed siRNA or the negative control (a “scrambled” sequence with no significant homology to any known gene sequences from mouse, rat or human cell lines) were used. Ten nM siRNA (scrambled or experimental) was diluted in serum-free media. Four μl Hiperfect (Qiagen) was added to this mixture and the solution was mixed by pipetting up and down several times. This solution was incubated at room temp for 10 min, then added dropwise to each dish. The medium in each dish was swirled gently to mix, then incubated at 37° C for 2h. Serum-containing medium was added to each plate, and cells were incubated at 37° C for 24h before then treated with drugs (0-24h).

### Assessments of autophagosome and autolysosome levels

Autophagy studies made use of a plasmid which produces an LC3-GFP-RFP fusion protein. In autophagosomes, both GFP and RFP fluoresce whereas in the acidic autolysosome only RFP fluoresces. Transfected cells expressing LC3-GFP-RFP, were, as indicated, also transfected with siRNA molecules. After an additional 24h, cells were treated with vehicle control or with the test agents as shown in each graph. Cells were visualized at 60X magnification after 4 h and 8 h of drug exposure. At least fifty randomly selected cells are examined and the mean number of GFP+ RFP+ and RFP+ only punctae per cell determined. Three independent triplicates from separate wells used to calculate the mean number of punctae per cell.

### Data analysis

Comparison of the effects of various treatments was using one-way ANOVA for normalcy followed by a two tailed Student’s t-test with multiple comparisons. Differences with a p-value of < 0.05 were considered statistically significant. Experiments are the means of multiple individual data points per experiment from 3 independent experiments (± SD). Data in each Figure has statistical annotation with the actual standard deviation value removed for clarity.

### Data availability

Upon appropriate request, data will be shared with others.

## RESULTS

**Figure 2:**
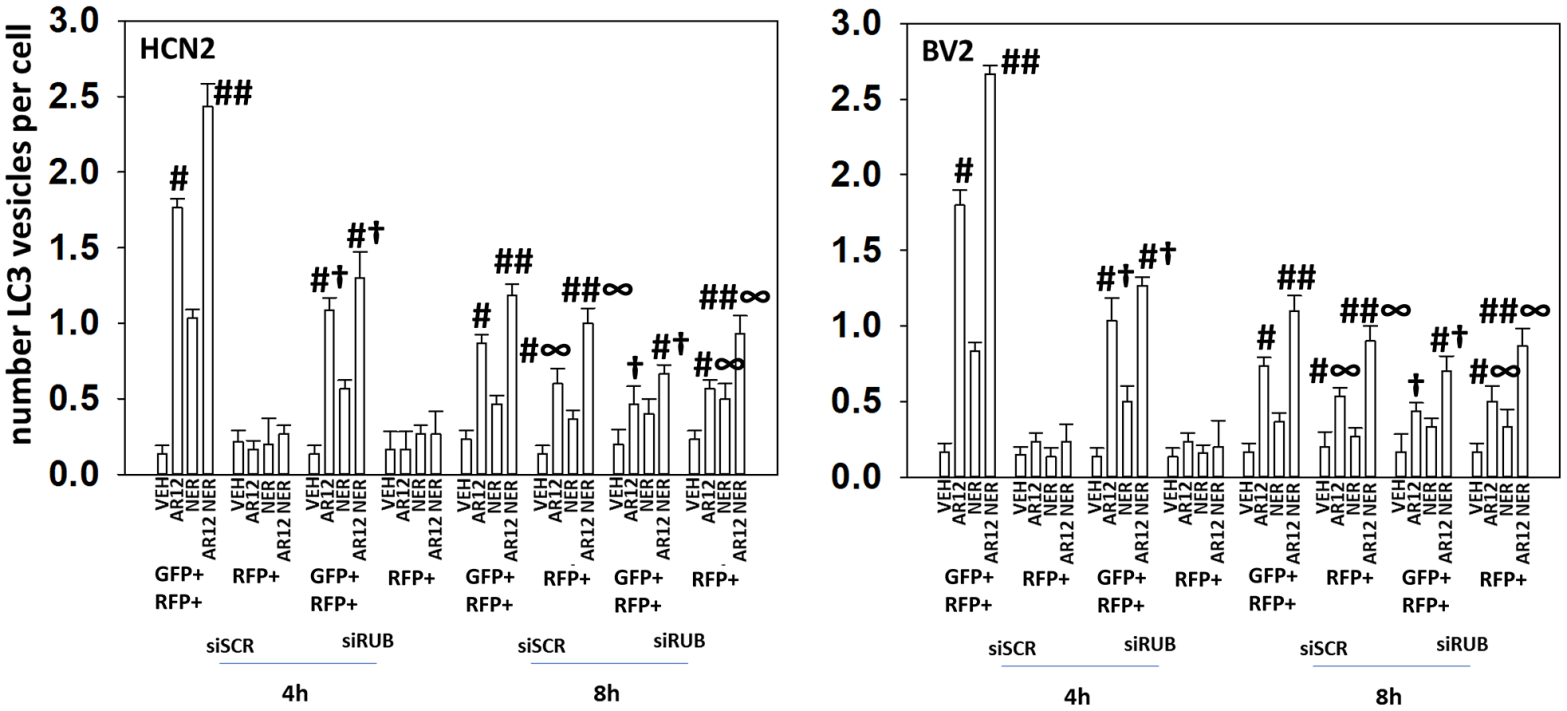
**Knock down of Rubicon suppresses drug-induced autophagosome formation but does not appear to alter autophagic flux.** HCN2 neuronal cells and BV2 microglial cells were transfected with a scrambled siRNA or with an siRNA to knock down the expression of Rubicon and were co-transfected with a plasmid to express LC3-GFP-RFP. After 24h, cells were treated with vehicle control, AR12 (2 μM), neratinib (50 nM) or the drugs in combination for 4h and 8h. The mean number of intense GFP+RFP+ and RFP+ punctae per cell was determined (n = 3 +/-SD) # p < 0.05 greater than vehicle control; ## p < 0.05 greater than AR12 alone value; † p < 0.05 less than corresponding value in siSCR cells; ∞ p < 0.05 greater than corresponding value at the 4h timepoint.

**Figure 3:**
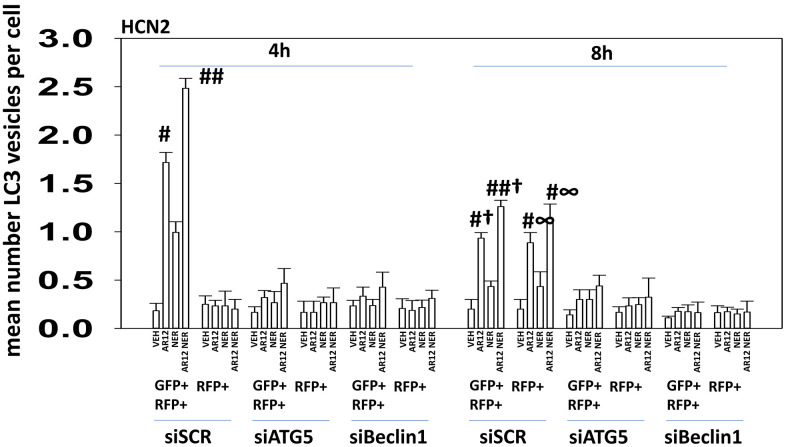
**Knock down of Beclin1 or ATG5 prevents autophagosome formation and autophagic flux in HCN2 cells.** HCN2 neuronal cells were transfected with a scrambled siRNA or with an siRNA to knock down the expression of ATG5 or Beclin1 and were co-transfected with a plasmid to express LC3-GFP-RFP. After 24h, cells were treated with vehicle control, AR12 (2 μM), neratinib (50 nM) or the drugs in combination for 4h and 8h. The mean number of intense GFP+RFP+ and RFP+ punctae per cell was determined (n = 3 +/-SD) # p < 0.05 greater than vehicle control; ## p < 0.05 greater than AR12 alone value; † p < 0.05 less than corresponding value in siSCR cells; ∞ p < 0.05 greater than corresponding value at the 4h timepoint.

The chaperone GRP78 acts both as a chaperone to renature proteins but also plays a pivotal role in the abilities of cells to sense endoplasmic reticulum (ER) stress [15]. GRP78 is located both in the ER and on the outer leaflet of the plasma membrane. In the ER, GRP78 inhibits PKR-like endoplasmic reticulum kinase (PERK), which phosphorylates and inactivates eIF2α on S51. On the cell surface GRP78 plays roles in stabilizing plasma membrane receptors and more recently was shown to play an essential role as a co-receptor for the virus SARS-CoV-2 [9, 16]. We have previously shown that neratinib, via Rubicon-dependent LC3-associated phagocytosis (LAP), caused the internalization and subsequent macroautophagic degradation of growth factor receptors and RAS proteins [12–14]. In microglia, the uptake and degradation of APP has also been linked to LC3-associated endocytosis (LANDO) [24, 25]. Our present studies were designed to determine whether LAP / LANDO played a mechanistic role in the abilities of AR12 and neratinib to cause Tau, APP, and chaperone degradation in neurons and microglia. In HCN2 human neuronal cells and BV2 murine microglial cells, knock down of the essential LAP regulatory protein Rubicon suppressed autophagosome formation though did not significantly alter autophagic flux, i.e., vesicles that were initially GFP+ RFP+ became over time only RFP+ (Figure 2). Knock down of the macroautophagy proteins Beclin1 or ATG5 also abolished autophagosome formation and autophagic flux (Figure 3) [10].

**Figure 4:**
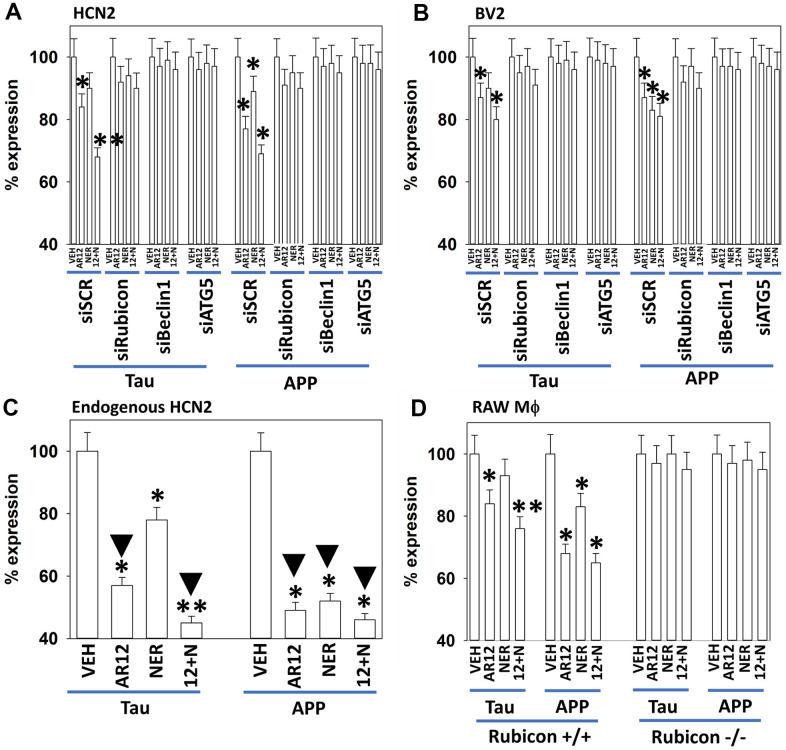
**Degradation of APP and Tau by AR12 and neratinib requires LAP and macroautophagy in HCN2 neuronal cells and in BV2 microglia.** (**A**, **B**) HCN2 cells were transfected with a scrambled siRNA or with siRNA molecules to knock down expression of Rubicon, Beclin1 or ATG5. In parallel, cells were transfected with an empty vector plasmid in (**B**), or transfected with plasmids to express APP or Tau in (**A**). After 24h, cells were treated with vehicle control, AR12 (2 μM), neratinib (50 nM) or the drugs in combination for 6h. Cells were fixed in place and immunostaining performed to determine the expression of Tau, APP and ERK2. (n = 3 +/-SD) Endogenous expression of APP was 5% of the value for APP expressed from a plasmid. Endogenous expression of Tau was 6% of the value for Tau expressed from a plasmid. * p < 0.05 less than vehicle control; ** p < 0.05 less than corresponding AR12 value; **▼**p < 0.05 greater degradation than corresponding value in cells transfected to express APP or Tau. (**C**) BV2 microglial cells were transfected with a scrambled siRNA or with siRNA molecules to knock down expression of Rubicon, Beclin1 or ATG5. In parallel, cells were transfected with plasmids to express APP or Tau. After 24h, cells were treated with vehicle control, AR12 (2 μM), neratinib (50 nM) or the drugs in combination for 6h. Cells were fixed in place and immunostaining performed to determine the expression of Tau, APP and ERK2. (n = 3 +/-SD). * p < 0.05 less than vehicle control. (**D**) RAW macrophages (+/+ and -/- for Rubicon) were transfected to express Tau or APP. After 24h, cells were treated with vehicle control, AR12 (2 μM), neratinib (50 nM) or the drugs in combination for 6h. Cells were fixed in place and immunostaining performed to determine the expression of Tau, APP and ERK2. * p < 0.05 less than vehicle control; ** p < 0.05 less than corresponding neratinib value.

Based on the data in Figures 2, 3, HCN2 neuronal and BV2 microglial cells were transfected with plasmids to express Tau or APP, and co-transfected with siRNA molecules to knock down the expression of Rubicon, Beclin1 or ATG5. AR12 and neratinib reduced the expression of Tau and APP in HCN2 cells, that was blocked by knock down of Rubicon, Beclin1 or ATG5 (Figure 4A). Knock down of Rubicon, Beclin1 or ATG5 did not significantly alter the basal expression levels of Tau or APP (not shown). Knock down of Rubicon, Beclin1 or ATG5 blocked the degradation of Tau and APP in BV2 microglia (Figure 4B). ompared to the amount of APP expressed from a transfected plasmid (100%), endogenous APP expression in the HCN2 cells was only 5%. For Tau expression, it was 6%. AR12 and neratinib, to a significantly greater extent than observed when expressing Tau or APP from plasmids, reduced endogenous Tau and APP levels in HCN2 cells (Figure 4C). To confirm our Rubicon siRNA data, we made use of RAW macrophages that had been genetically deleted for Rubicon. In wild type RAW macrophages, AR12 and neratinib reduced the expression of chaperones, Tau, and APP, and increased eIF2α S51 phosphorylation (Figure 4D). Deletion of Rubicon in the macrophages abolished the degradation of all tested proteins and the increase in eIF2α S51 phosphorylation (Supplementary Figure 1).

**Figure 5:**
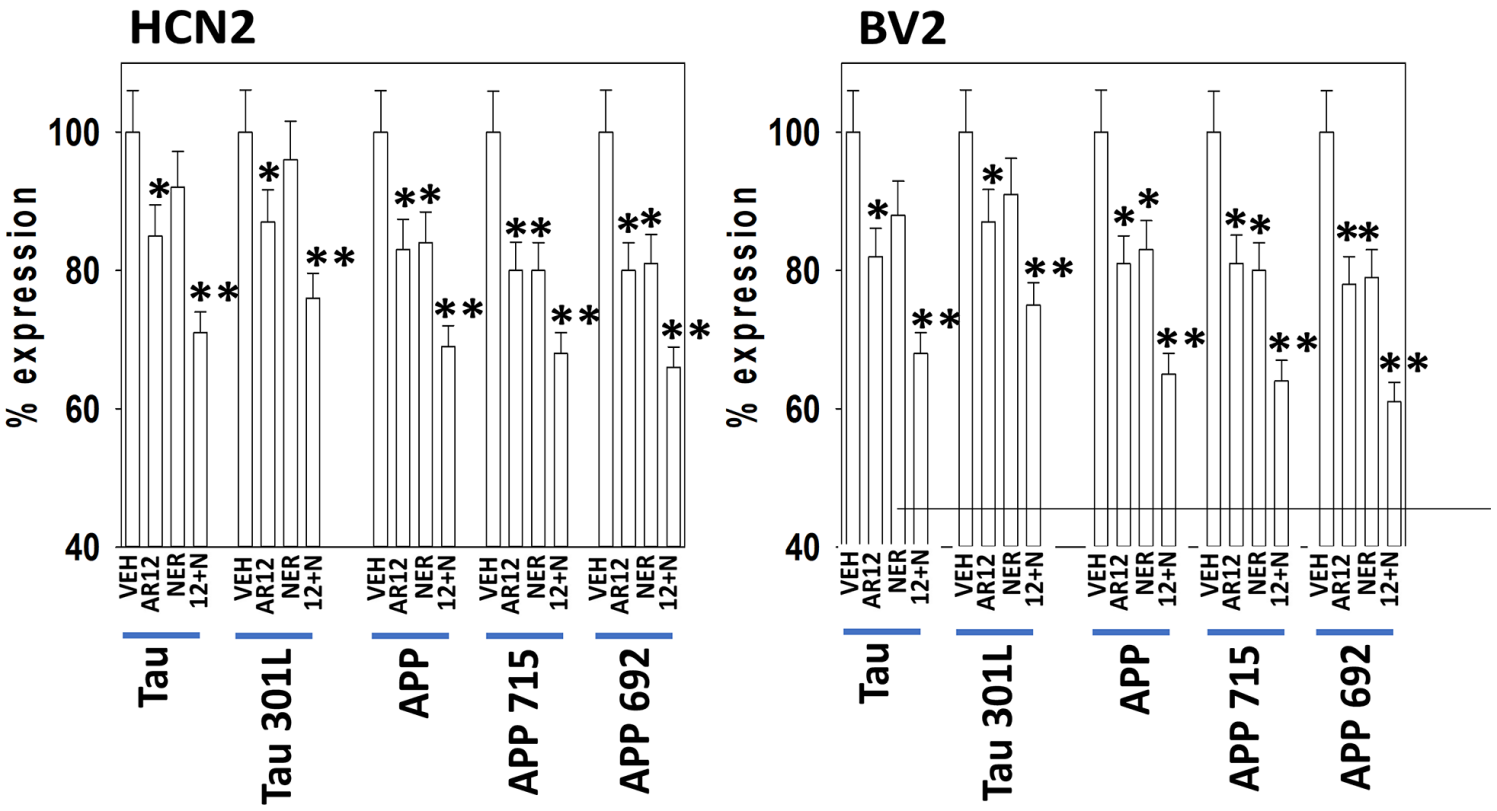
**AR12 and the drug combination reduce the expression of mutant forms of TAU and APP.** HCN2 and BV2 cells were transfected with plasmids to express wild type TAU or TAU 301L. After 24h, cells were treated with vehicle control, AR12 (2 μM), neratinib (50 nM) or the drugs in combination for 6h. Cells were fixed in place and immunostaining performed to determine the expression of TAU, TAU 301L and ERK2. (n = 3 +/-SD) * p < 0.05 less than vehicle control; ** p < 0.05 less than AR12 value. In parallel, HCN2 and BV2 cells were transfected with plasmids to express wild type APP or APP 715 or APP 692. After 24h, cells were treated with vehicle control, AR12 (2 μM), neratinib (50 nM) or the drugs in combination for 6h. Cells were fixed in place and immunostaining performed to determine the expression of APP, APP 715, APP 692 and ERK2. (n = 3 +/-SD) * p < 0.05 less than vehicle control; ** p < 0.05 less than AR12 value.

We next determined in HCN2 neuronal cells and in BV2 microglia the abilities of AR12 and neratinib to reduce the expression of mutant forms of APP and Tau [26–29]. AR12 and the drug combination reduced the expression of Tau 301L which trended to be less than the reduction of wild type Tau (Figure 5). AR12 and the drug combination was equipotent at reducing the expression of APP, APP715 and APP 692. These findings are important for future *in vivo* studies as, for example, the Tau P301L mutant is used in transgenic models of Alzheimer’s Disease.

**Figure 6:**
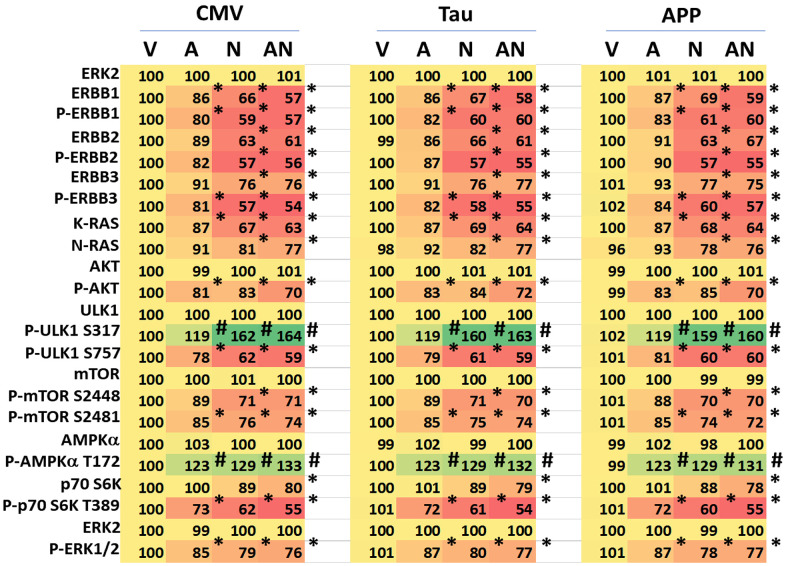
**Expression of Tau or APP does not significantly alter the regulation of protein phosphorylation or protein expression caused by AR12 and neratinib.** HCN2 cells were transfected with an empty vector plasmid or with plasmids to express Tau or APP. Twenty-four h afterwards, cells were treated with vehicle control, AR12 (2 μM), neratinib (50 nM) or the drugs in combination for 6h. Cells were fixed in place and immunostaining performed to determine the phosphorylation and expression of the indicated proteins (n = 3 +/-SD). * p < 0.05 less than vehicle control; # p < 0.05 greater than vehicle control. All expression / phosphorylation levels were normalized to vehicle control cells transfected with the empty vector plasmid.

**Figure 7:**
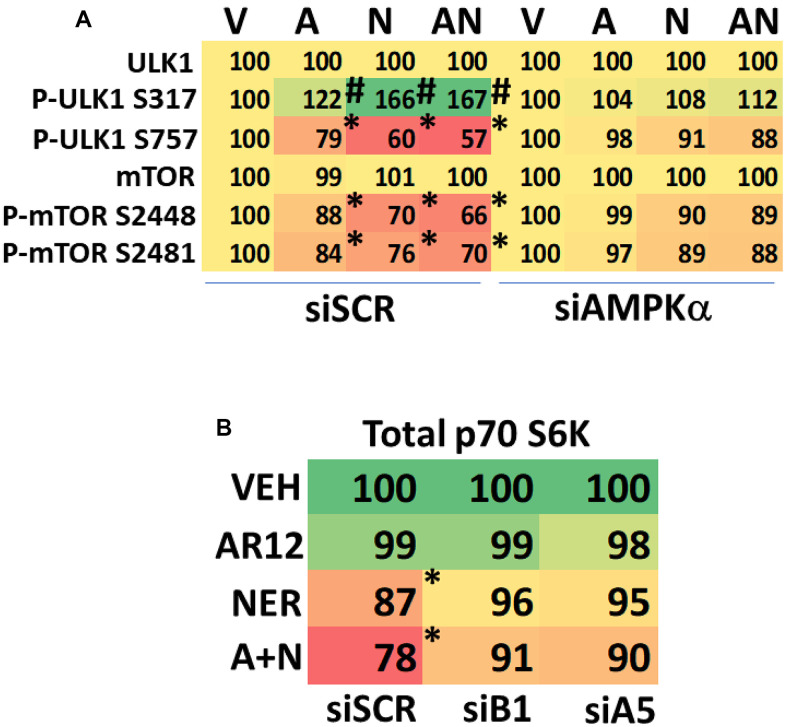
**AR12 and neratinib cause degradation of p70 S6K in HCN2 neuronal cells.** (**A**) HCN2 cells were transfected with a scrambled siRNA control or with an siRNA to knock down expression of AMPKa. After 24h, cells were treated with vehicle control or with [AR12 (2 μM) + neratinib (50 nM)] for 6h. Cells were fixed in place and immunostaining performed to determine the phosphorylation and expression of the indicated proteins (n = 3 +/-SD). * p < 0.05 less than vehicle control; # p < 0.05 greater than vehicle control. (**B**) HCN2 cells were transfected with a scrambled siRNA control or with siRNA molecules to knock down expression of either Beclin1 or ATG5. After 24h, cells were treated with vehicle control or with [AR12 (2 μM) + neratinib (50 nM)] for 6h. Cells were fixed in place and immunostaining performed to determine the phosphorylation and expression of the indicated proteins (n = 3 +/-SD). * p < 0.05 less than vehicle control; # p < 0.05 greater than vehicle control.

We hypothesized that expression of Tau or APP may alter the behavior of intracellular signaling pathways when cells are treated with AR12 and neratinib. HCN2 cells were treated with AR12 and neratinib for 6h, after which alterations in protein expression and protein phosphorylation were determined. As we have observed in other cell types, neratinib reduced both the expression and the phosphorylation of the plasma membrane receptors ERBB1/2/3 (Figure 6). Neither expression of Tau nor expression of APP significantly altered the levels of drug-induced protein degradation or protein phosphorylation when compared to empty vector transfected cells. Notably, compared to tumor cell types we have previously treated with neratinib as a single agent, we observed a profound increase in the phosphorylation of ULK1 S317 and profound reductions in the phosphorylation of ULK1 S757, mTORC1 S2448, mTORC2 S2481 and p70 S6K T389. Increased ULK1 S317 phosphorylation concomitant with lower ULK1 S757 phosphorylation results in a very high level of ULK1 catalytic activity which drives autophagosome formation. Knock down of AMPKα prevented the alterations in protein phosphorylation observed in mTOR and ULK1 (Figure 7A). Expression of an activated mTOR protein suppressed autophagosome formation and the degradation of APP and Tau (not shown). The most surprising data was that AR12 and neratinib combined to not only reduce p70 S6K T389 phosphorylation but also to reduce p70 S6K protein levels. Knock down of the macroautophagy regulatory proteins Beclin1 or ATG5 prevented p70 S6K degradation (Figure 7B). As p70 S6K signaling has been linked to enhanced Tau phosphorylation, we hypothesize that the portions of p70 S6K complexed with Tau were being degraded by macroautophagy in our cells.

**Figure 8:**
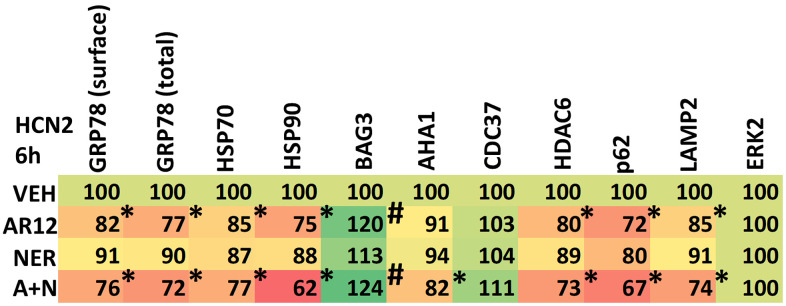
**AR12 and neratinib reduce chaperone expression in neuronal cells.** HCN2 neuronal cells were treated with vehicle control, AR12 (2 μM), neratinib (50 nM) or the drugs in combination for 6h. Cells were fixed in place and immunostaining performed to determine the expression of GRP78 (total and cell surface), HSP70, HSP90, eIF2α and ERK2, and the phosphorylation of eIF2α S51. (n = 3 +/-SD) * p < 0.05 less than vehicle control; # p < 0.05 greater than vehicle control.

**Figure 9:**
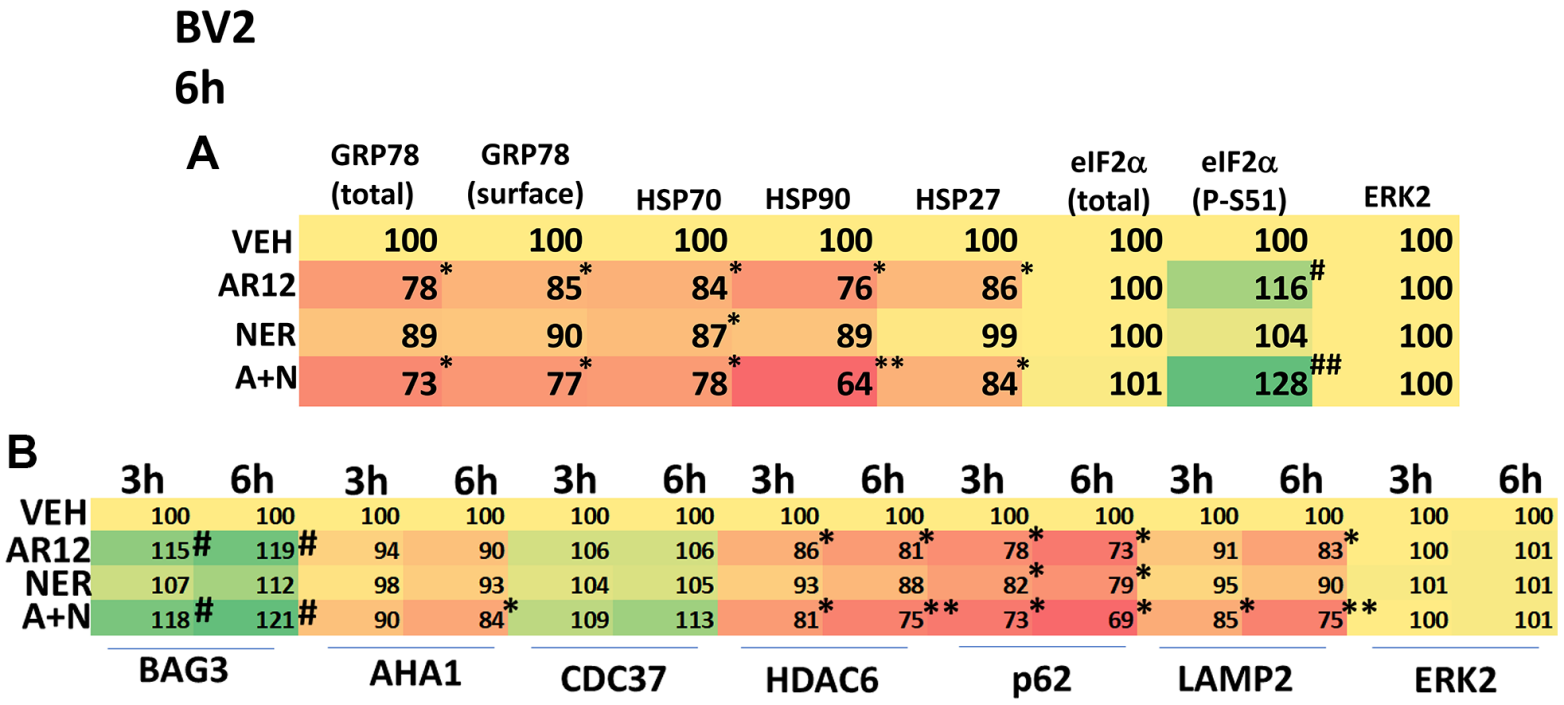
**AR12 and neratinib reduce chaperone expression and enhance ER stress signaling in microglia.** (**A**) BV2 microglial cells were treated with vehicle control, AR12 (2 μM), neratinib (50 nM) or the drugs in combination for 6h. Cells were fixed in place and immunostaining performed to determine the expression of GRP78 (total and cell surface), HSP70, HSP90, eIF2α and ERK2, and the phosphorylation of eIF2α S51. (n = 3 +/-SD) * p < 0.05 less than vehicle control; ** p < 0.05 less than AR12 treatment alone; # p < 0.05 greater than vehicle control; ## p < 0.05 greater than AR12 treatment alone. (**B**) BV2 microglial cells were treated with vehicle control, AR12 (2 μM), neratinib (50 nM) or the drugs in combination for 3h and 6h. Cells were fixed in place and immunostaining performed to determine the expression of BAG3, AHA1, CDC37, HDAC6, p62, LAMP2 and ERK2. (n = 3 +/-SD) * p < 0.05 less than vehicle control; ** p < 0.05 less than AR12 treatment alone; # p < 0.05 greater than vehicle control.

A key AR12 target are the ATP binding domains of HSP90 and HSP70 family chaperone proteins [3–10]. Both AR12 and neratinib can also reduce the protein levels of chaperone proteins by stimulating autophagy. In HCN2 neuronal and BV2 microglial cells, AR12 reduced the expression of GRP78 (cell surface and total), HSP70 and HSP90 (Figures 8, 9). AR12 and neratinib interacted to further reduce the expression of HSP90 and to inactivate eIF2α. Chaperones are complexed with other proteins including BAG3 (associated with HSP70) and AHA1 and CDC37 (associated with HSP90). BAG3, AHA1 and CDC37 have all been linked to AD pathology [30–36]. BAG3 has been shown to enhance Tau degradation by autophagy [24, 25, 30]. HSP90 and AHA1 promote Tau pathogenesis [31]. And CDC37 with HSP90 also acts to maintain Tau stability [34–36]. AR12 alone as well as the drug combination increased BAG3 expression (Figures 8, 9). The drug combination reduced AHA1 levels but did not alter the expression of CDC37. The histone deacetylase HDAC6 regulates HSP90 activity; increased HSP90 acetylation reduces chaperone function [37]. AR12 and the drug combination reduced HDAC6 expression, which will result in increased HSP90 acetylation concomitant with a further reduction in overall HSP90 chaperoning activity (Figures 8, 9).

**Figure 10:**
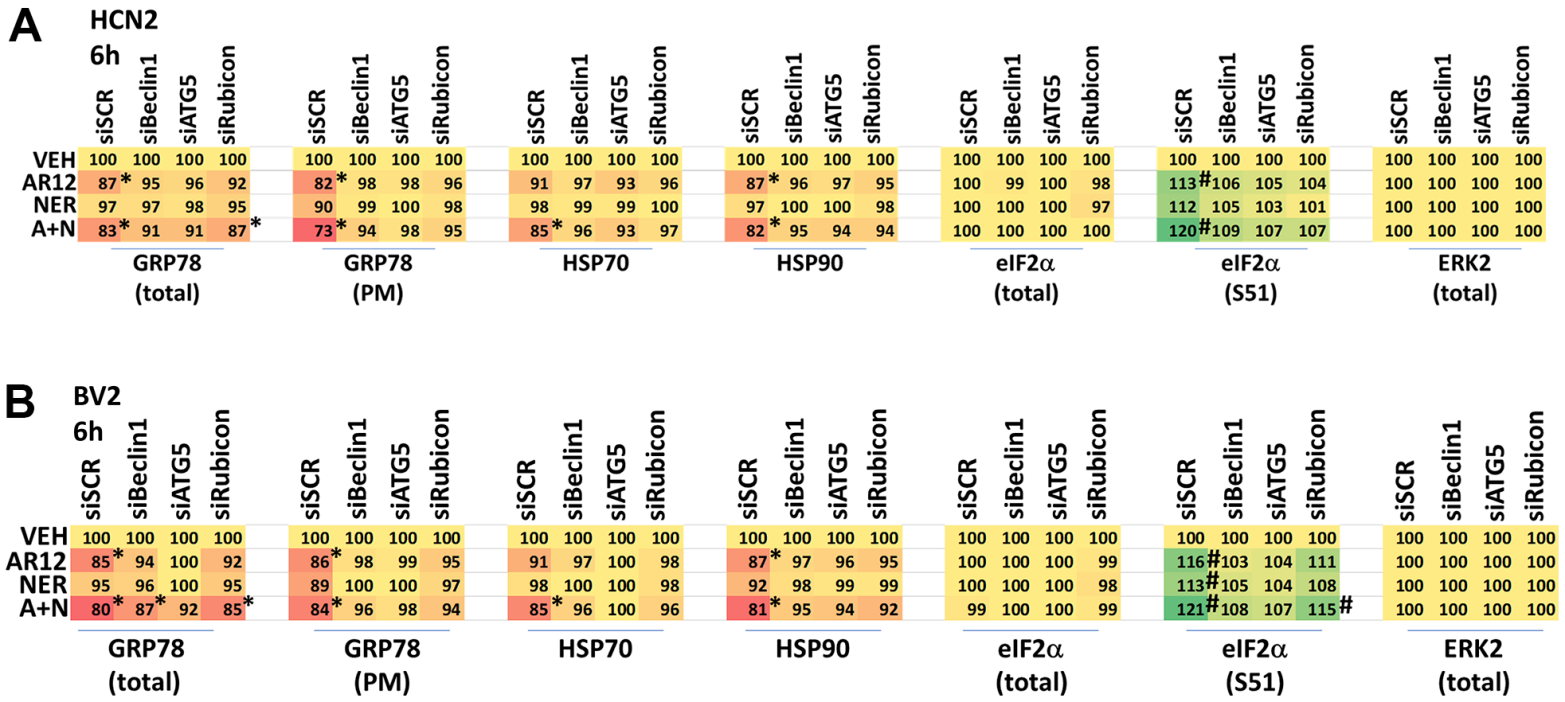
**Degradation of chaperones and eIF2α S51 phosphorylation requires LAP and macroautophagy.** (**A**) HCN2 and (**B**) BV2 cells were transfected with a scrambled siRNA or with siRNA molecules to knock down the expression of Rubicon, Beclin1 or ATG5. After 24h, cells were treated with vehicle control, AR12 (2 μM), neratinib (50 nM) or the drugs in combination for 6h. Cells were fixed in place and immunostaining performed to determine the expression of GRP78 (total and cell surface / plasma membrane), HSP70, HSP90, eIF2α and ERK2, and the phosphorylation of eIF2α S51. (n = 3 +/-SD) * p < 0.05 less than vehicle control; # p < 0.05 greater than vehicle control.

**Figure 11:**
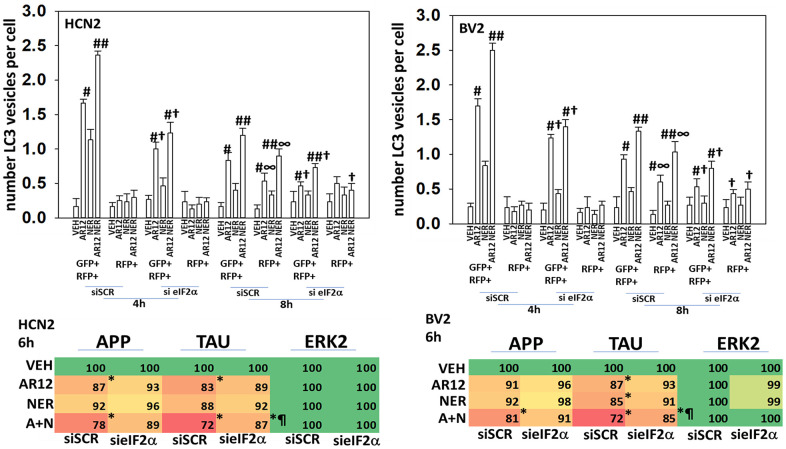
**ER stress signaling plays a key role in facilitating autophagy and protein degradation in neuronal cells.** Upper Graphs. HCN2 cells and BV2 cells were transfected with a scrambled siRNA or with an siRNA to knock down the expression of eIF2α and were co-transfected with a plasmid to express LC3-GFP-RFP. After 24h, cells were treated with vehicle control, AR12 (2 μM), neratinib (50 nM) or the drugs in combination for 4h and 8h. The mean number of intense staining GFP+RFP+ and RFP+ punctae per cell was determined (n = 3 +/-SD) # p < 0.05 greater than vehicle control; ## p < 0.05 greater than AR12 alone value; † p < 0.05 less than corresponding value in siSCR cells; ∞ p < 0.05 greater than corresponding value at the 4h timepoint. Lower Tables. HCN2 cells were transfected with plasmids to express Tau or APP and co-transfected with a scrambled siRNA or with an siRNA molecule to knock down the expression of eIF2α. After 24h, cells were treated with vehicle control, AR12 (2 μM), neratinib (50 nM) or the drugs in combination for 6h. Cells were fixed in place and immunostaining performed to determine the expression of Tau, APP and ERK2. (n = 3 +/-SD) * p < 0.05 less than vehicle control; ¶ p < 0.05 greater than corresponding value in siSCR cells.

**Figure 12:**
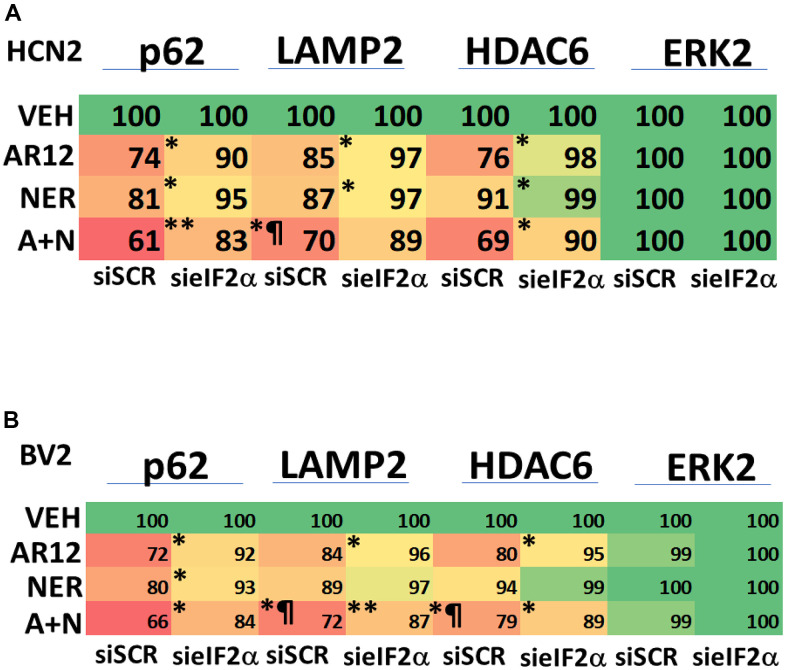
**ER stress signaling plays a key role in facilitating autophagy and HDAC6 protein degradation.** (**A**) HCN2 cells were transfected with a scrambled siRNA or with an siRNA molecule to knock down the expression of eIF2α. After 24h, cells were treated with vehicle control, AR12 (2 μM), neratinib (50 nM) or the drugs in combination for 6h. Cells were fixed in place and immunostaining performed to determine the expression of HDAC6, LAMP2, p62 and ERK2. (n = 3 +/-SD) * p < 0.05 less than vehicle control; ** p < 0.05 less than either of the individual treatments; ¶ p < 0.05 greater than corresponding value in siSCR cells. (**B**) BV2 cells were transfected with a scrambled siRNA or with an siRNA molecule to knock down the expression of eIF2α. After 24h, cells were treated with vehicle control, AR12 (2 μM), neratinib (50 nM) or the drugs in combination for 6h. Cells were fixed in place and immunostaining performed to determine the expression of HDAC6, LAMP2, p62 and ERK2. (n = 3 +/-SD) * p < 0.05 less than vehicle control; ** p < 0.05 less than either of the individual treatments; ¶ p < 0.05 greater than corresponding value in siSCR cells.

In neuronal cells and microglia, knock down of Rubicon, Beclin1 or ATG5 prevented the drugs alone or in combination from reducing the expression of chaperone proteins (Figures 10A, 10B). Knock down of Beclin1 or ATG5 prevented AR12 alone or in combination from enhancing eIF2α S51 phosphorylation in microglia whereas knock down of Rubicon did not (Figure 10B) [38–41]. We discovered, however, that knock down of eIF2α significantly reduced autophagosome formation and prevented the degradation of Tau and APP by AR12 and neratinib (Figure 11). This implies eIF2α regulates autophagy, but that autophagy also regulates serine 51 phosphorylation of eIF2α. In agreement with the autophagy data in Figure 11, AR12 and the drug combination reduced expression of p62 and LAMP2 (Figure 12). Thus, we hypothesize that the initial inactivation of GRP78 catalytic function by AR12 facilitates an initial increase in eIF2α phosphorylation which in turn is essential for greater levels of eIF2α phosphorylation, greater levels of macroautophagy and eventually leading to significant amounts of Tau / APP / chaperone protein degradation.

**Figure 13:**
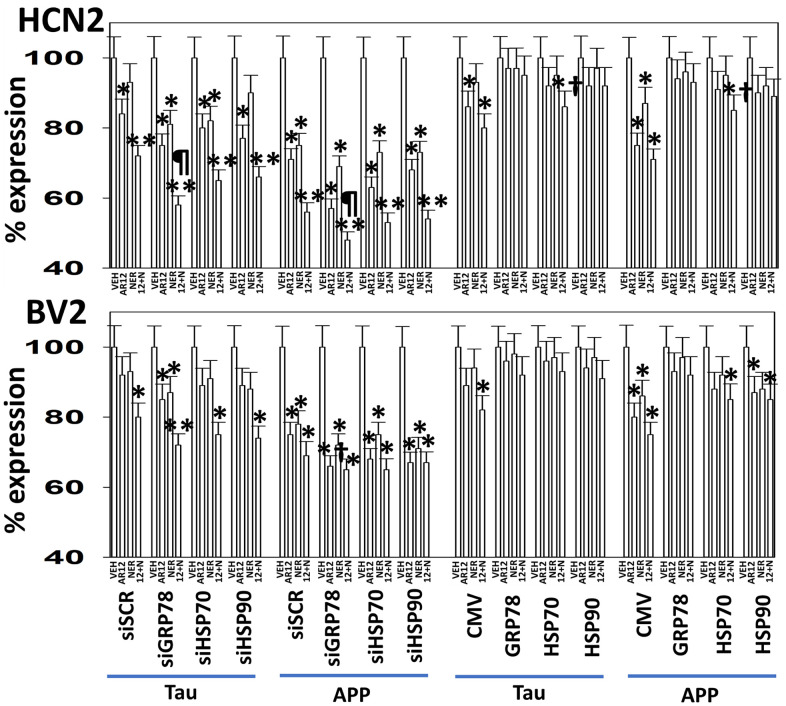
**GRP78 plays a key role in regulating the expression of APP and Tau after exposure of neuronal cells and microglia to AR12 and neratinib.** HCN2 and BV2 cells were transfected with a scrambled siRNA or with siRNA molecules to knock down the expression of GRP78, HSP70 or HSP90 and in parallel co-transfected to express Tau or APP. After 24h, cells were treated with vehicle control, AR12 (2 μM), neratinib (50 nM) or the drugs in combination for 6h. Cells were fixed in place and immunostaining performed to determine the expression of APP, Tau and ERK2. (n = 3 +/-SD) * p < 0.05 less than vehicle control; ** p < 0.05 less than AR12 value; ¶ p < 0.05 less than corresponding value in siSCR cells. In parallel, HCN2 and BV2 cells were transfected with an empty vector plasmid CMV or with plasmids to express GRP78, HSP70 or HSP90 and in parallel co-transfected to express Tau or APP. After 24h, cells were treated with vehicle control, AR12 (2 μM), neratinib (50 nM) or the drugs in combination for 6h. Cells were fixed in place and immunostaining performed to determine the expression of APP, Tau and ERK2. (n = 3 +/-SD) * p < 0.05 less than vehicle control; ** p < 0.05 less than AR12 value; † p < 0.05 greater than corresponding value in siSCR cells.

Based on our data showing reduced chaperone expression following drug exposure, we next defined which chaperones played the most important roles in regulating APP and Tau stability; in HCN2 and BV2 cells (Figure 13); in RAW macrophages (Supplementary Figure 2). Over-expression of GRP78, HSP70 or HSP90, or knock down of GRP78, HSP70 or HSP90 surprisingly did not significantly alter the basal expression levels of APP and Tau (not shown). This data demonstrates that over-expression of either GRP78, HSP70 or HSP90 prevented the drug-induced degradation of APP. Over-expression of GRP78, but not of HSP70 or HSP90, prevented the drug combination from reducing Tau expression. Knock down of GRP78, but not of HSP70 or HSP90, enhanced the ability of AR12 alone and the drug combination to reduce APP expression. Knock down of GRP78 also further enhanced the ability of AR12 as a single agent to reduce Tau levels. Our GRP78 data is congruent with our earlier findings when knocking down eIF2α expression. Collectively these findings strongly argue that the chaperone GRP78 and translation regulator eIF2α play key roles in regulating the ability of AR12 and neratinib to reduce Tau and APP protein levels.

**Figure 14:**
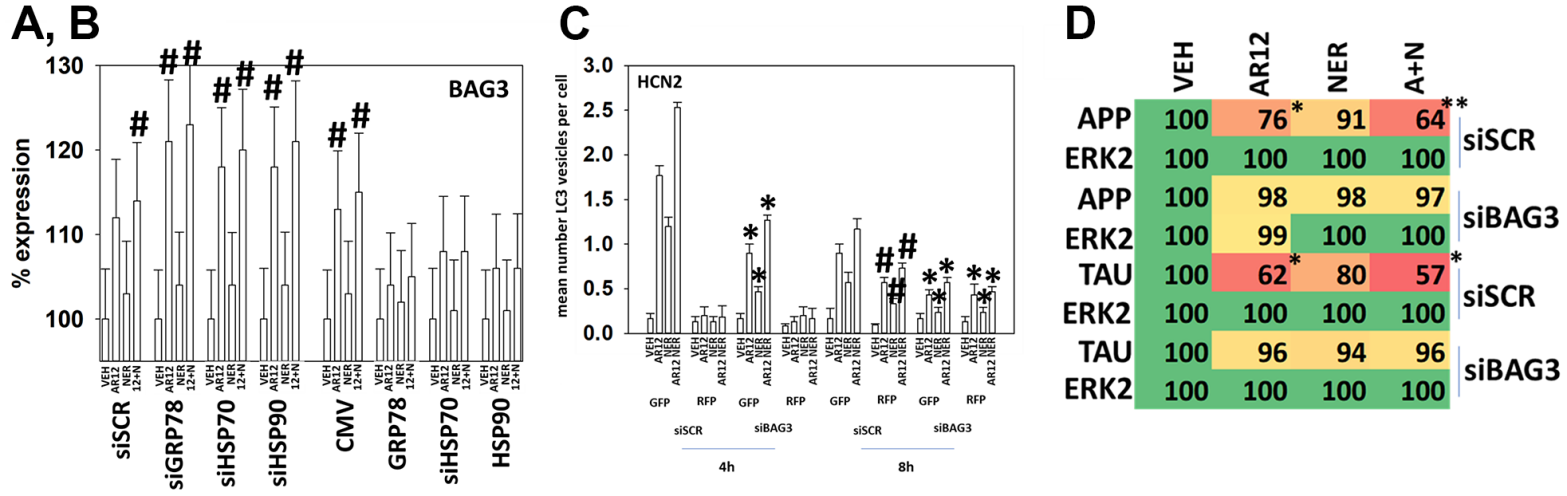
**Over-expression of GRP78 suppresses the drug-induced expression of BAG3.** (**A**) HCN2 cells were transfected with a scrambled siRNA or with siRNA molecules to knock down the expression of GRP78, HSP70 or HSP90. After 24h, cells were treated with vehicle control, AR12 (2 μM), neratinib (50 nM) or the drugs in combination for 6h. Cells were fixed in place and immunostaining performed to determine the expression of BAG3 and ERK2. (n = 3 +/-SD) # p < 0.05 greater than vehicle control; † p < 0.05 less than corresponding value in CMV cells. (**B**) HCN2 cells were transfected with an empty vector plasmid or with plasmids to over-express GRP78, HSP70 or HSP90. After 24h, cells were treated with vehicle control, AR12 (2 μM), neratinib (50 nM) or the drugs in combination for 6h. Cells were fixed in place and immunostaining performed to determine the expression of BAG3 and ERK2. (n = 3 +/-SD) # p < 0.05 greater than vehicle control; † p < 0.05 less than corresponding value in CMV cells. (**C**) HCN2 cells were transfected with a scrambled siRNA or an siRNA to knock down BAG3 expression. In parallel, they were transfected with a plasmid to express LC3-GFP-RFP. After 24h, cells were treated with vehicle control, AR12 (2 μM), neratinib (50 nM) or the drugs in combination for 4h and 8h. The mean number of intense GFP+RFP+ and RFP+ punctae per cell were determined (n = 3 +/-SD) * p < 0.05 less than corresponding siSCR value; # p < 0.05 greater than corresponding value at 4h. (**D**) HCN2 cells were transfected with a scrambled siRNA or with an siRNA to knock down BAG3 expression. In parallel, they were transfected with plasmids to express either Tau or APP. After 24h, cells were treated with vehicle control, AR12 (2 μM), neratinib (50 nM) or the drugs in combination for 6h. Cells were fixed in place and immunostaining performed to determine the expression of Tau, APP and ERK2. (n = 3 +/-SD) * p < 0.05 less than vehicle control; ** p < 0.05 less than corresponding AR12 value.

The co-chaperone BAG3 has been shown to facilitate the degradation of Tau and APP [30–32]. Over-expression of GRP78, HSP70 or HSP90, or knock down of GRP78, HSP70 or HSP90 did not alter the basal expression level of BAG3 (not shown). Knock down of GRP78, HSP70 or HSP90 significantly enhanced the ability of AR12 to enhance BAG3 expression (Figure 14A, 14B). Over-expression of GRP78, HSP70 or HSP90 significantly reduced the ability of AR12, alone or in combination with neratinib, from enhancing BAG3 levels (Figure 14A, 14B). Thus, reduced chaperone levels facilitate more drug-induced BAG3 expression.

**Figure 15:**
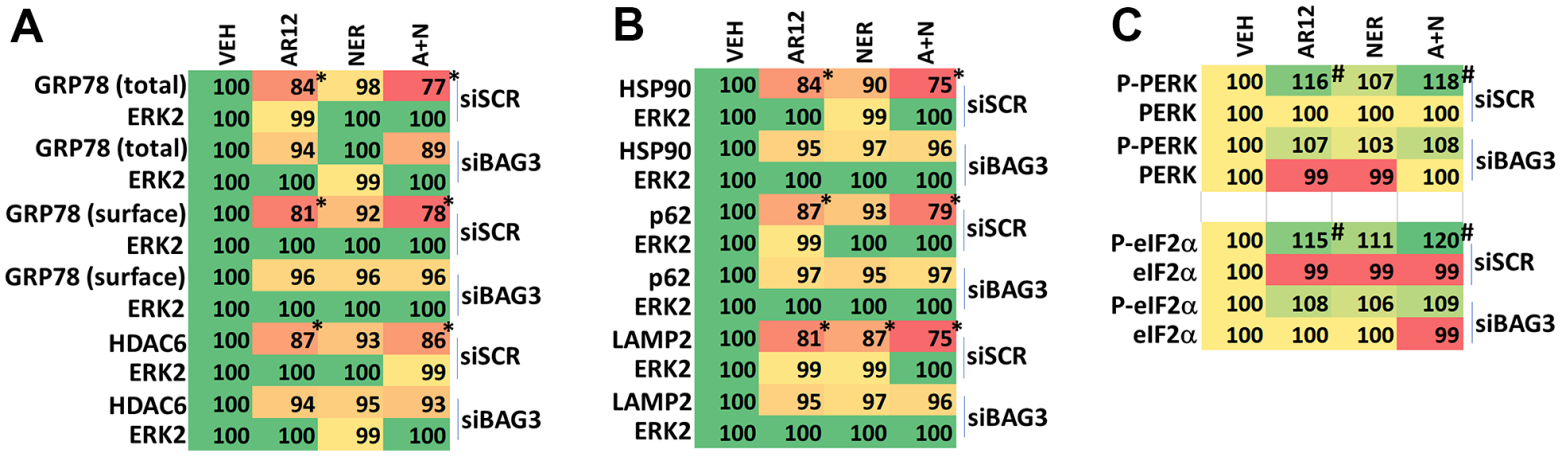
**BAG3 is essential for drug-induced degradation of GRP78.** (**A**–**C**) HCN2 cells were transfected with a scrambled siRNA or with an siRNA to knock down BAG3 expression. After 24h, cells were treated with vehicle control, AR12 (2 μM), neratinib (50 nM) or the drugs in combination for 6h. Cells were fixed in place and immunostaining performed to determine the expression of GRP78 (total and cell surface), HDAC6, HSP90, p62, LAMP2, PERK, P-PERK T980, eIF2α and P-eIF2α S51 and ERK2. (n = 3 +/-SD) * p < 0.05 less than vehicle control; ** p < 0.05 less than corresponding AR12 value; # p < 0.05 greater than vehicle control.

Knock down of BAG3 reduced drug-induced autophagosome formation and autophagic flux (Figure 14C). AR12 and neratinib, as previously observed, profoundly reduced the expression of Tau and APP, and knock down of BAG3 almost abolished the abilities of AR12 and neratinib to cause degradation of Tau (~92%) and APP (~91%) (Figure 14D). Knock down of BAG3 also significantly reduced the abilities of AR12 and neratinib to reduce the expression of GRP78 (total and cell surface) and of HDAC6 (Figure 15A). Notably and in contrast to Tau and APP, a trend of GRP78 degradation was observed even in drug-treated BAG3 knock down cells, with a reduction in degradation of only ~48%. This data suggests that the regulation of APP and Tau expression after AR12 / neratinib exposure, compared to GRP78, is exquisitely dependent upon BAG3 expression.

Knock down of BAG3 prevented the degradation of HSP90, p62 and LAMP2 which is congruent with our autophagy data (Figure 15B). AR12 and neratinib activated PERK and significantly increased eIF2α S51 phosphorylation (Figure 15C). Knock down of BAG3 reduced the ability of the drugs to increase PERK and eIF2α phosphorylation. This data suggests that the initial catalytic inhibition of GRP78 by AR12 that facilitates ER stress signaling, which in turn leads to autophagy, and degradation of GRP78, acts in a feed-forward fashion to fully activated ER stress signaling.

**Figure 16:**
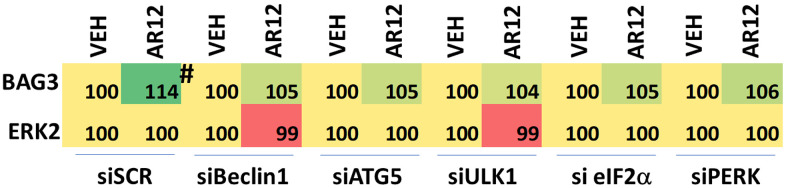
**Enhanced BAG3 expression requires autophagy and ER stress signaling.** HCN2 cells were transfected with a scrambled siRNA control or with siRNA molecules to knock down the expression of Beclin1, ATG5, ULK1, eIF2α or PERK. After 24h, cells were treated for 6h with vehicle control or with AR12 (2 μM). Cells were fixed in place and immunostaining performed to detect the expression of BAG3 and ERK2 (n = 3 +/-SD) # p < 0.05 greater than vehicle control.

Finally, we attempted to link cause-and-effect for the actions of AR12 upon the expression of BAG3 and the role of autophagy and ER stress signaling. Knock down of Beclin1, ATG5, ULK1, eIF2α or PERK significantly reduced AR12-induced BAG3 expression (Figure 16). This data further supports the concept that the drugs cause a feed-forward signaling loop via ER stress signaling and autophagy to degrade Tau and APP.

## DISCUSSION

**Figure 17:**
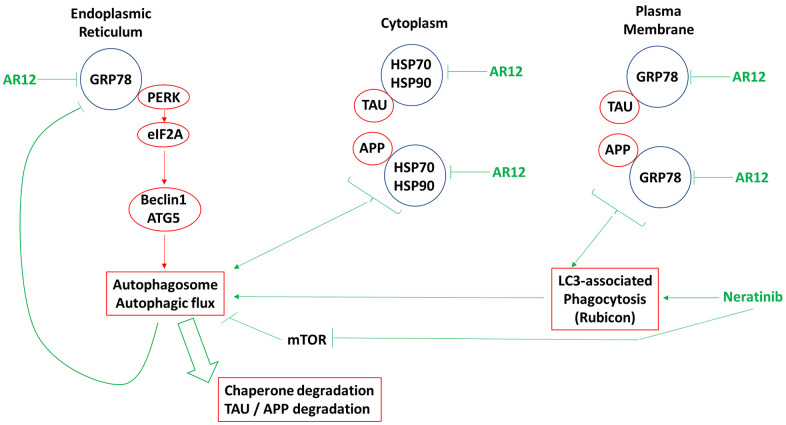
**The molecular mechanisms by which AR12 and neratinib act to degrade the expression of chaperones, Tau, and APP.** The initial action of AR12 is to inhibit multiple chaperone ATPase activities, particularly that of GRP78. This causes a modest increase in eIF2α phosphorylation in the endoplasmic reticulum which is responsible for modest increases in the expression of Beclin1 and ATG5. Neratinib, via regulation of small GTP binding proteins, receptors and MST4 causes LAP, resulting in the internalization of plasma membrane GRP78 and the proteins it chaperones, Tau and APP. Inhibition of cytosolic HSP90 and HSP70 also ultimately enhances ER stress signaling and autophagosome formation. Following the initial drug-induced signals that destabilize proteins and promote an ER stress / autophagy response, additional on-going degradation of GRP78 amplifies the initial response causing greater amounts of eIF2α phosphorylation, greater amounts of Beclin1 and ATG5 expression and significantly more autophagosome formation which is associated with autophagic flux. Thus, a self-supporting ER stress / autophagy response is generated that acts to further reduce Tau and APP expression.

The present studies were performed to determine whether AR12 and neratinib in microglia and neuronal cells caused the degradation of Tau and APP. Our data in a neuronal cell line, a microglial cell line and an established monocyte cell line, were near identical to our prior findings in HCT116 colon cancer cells. Previously we had shown that AR12 and neratinib reduced Tau and APP levels via macro-autophagy and that cells expressing the autophagy protein ATG16L1 T300 were more capable of autophagosome formation and APP / Tau degradation than cells expressing the ATG16L1 A300 isoform [10, 38–42]. In the present studies, knock down of a regulator of LC3-associated phagocytosis / endocytosis, Rubicon, significantly reduced the abilities of the drugs alone or in combination to reduce the expression of chaperone proteins, Tau and APP. LAP cooperated with macroautophagy in facilitating the degradation of Tau and APP (Figure 17) [43].

In our initial studies, we discovered that AR12 significantly increased BAG3 expression. BAG3 has been shown to play a role in the regulation of eIF2α phosphorylation and promote macroautophagy, HDAC6 function, and a transcriptional regulatory in its own right and with heat shock factor 1 (HSF1) [44–48]. The IRE1 ER stress pathway has been proposed to regulate the transcription of BAG3 in part by the regulation of HSF1 [48, 49]. Over-expression of GRP78 prevents both the phosphorylation of eIF2α and also activation of the IRE1 ER stress pathway and our data demonstrated that expression of GRP78 almost abolished the drug-induced expression of BAG3. This data argues we are inducing a coordinated series of cell signals which promote ER stress signaling to increase the expression of BAG3, Beclin1 and ATG5 which collectively facilitate the formation of autophagosomes which sequester chaperones, Tau, and APP, leading to their degradation.

GRP78 is localized in the ER where it binds to and inactivates PERK and chaperones newly synthesized proteins and in the outer leaflet of the plasma membrane where it mains stability of receptors and itself can act as a docking protein. AR12 reduced the protein levels of ER- and plasma membrane-localized GRP78 however the mechanisms by which this occurred were overlapping but not identical. The degradation of ER-localized required macroautophagy whereas the reduction in membrane-localized GRP78 required LAP and macroautophagy. The ability of the AR12 and neratinib drug combination to increase eIF2α S51 phosphorylation also reflected this pattern where knock down of Rubicon did not prevent eIF2α inactivation. In AD, the ability of cells to mount an ER stress response and clear denatured proteins is impaired and our findings argue that one way to overcome this issue is the application of AR12 and neratinib which complement each other in promoting ER stress signals and protein degradation.

Membrane localized GRP78 is capable of sensing the presence of denatured Tau and APP in the extracellular space liminal to the plasma membrane of neurons and microglia. Extracellular GRP78 chaperones both Tau and APP and extracellular GRP78 is essential for amyloid-β uptake by microglia [50–56]. Previously we noted that cells expressing ATG16L1 T300 expressed 25% higher cell surface levels of GRP78 than cells expressing ATG16L1 A300 [10]. Amyloid-β induces cells to over-express GRP78 and GRP78 is over-expressed in neurons from APP/PS1 mice [57, 58]. n.b. This is the well-described ER stress response that occurs after any perceived overload of denatured protein. The exogenous membrane-associated GRP78, once ingested with the amyloid-β / Tau proteins, was shown to translocate to the ER of the microglia where it acts to block ER stress signaling by PERK, and hence the macroautophagic digestion of denatured Tau and amyloid-β proteins. Thus, neurons and microglia from persons with more surface GRP78 are likely to have an enhanced capability to take up extracellular materials such as Tau and APP which, in contrast to the beneficial effect this has in Crohn’s Disease, in AD is deleterious. And, if Tau is considered to have ‘prion’-like properties, elevated plasma membrane GRP78 levels will result in a greater amount of Tau being propagated / seeded into bystander neurons and microglia.

Signaling by ERBB2 and KRAS play important roles in the development and progression of Alzheimer’s Disease [22, 23, 59–64]. Neratinib as a single agent and trending more so when combined with AR12 reduced both ERBB2 and KRAS levels. The robust changes in protein phosphorylation after drug exposure demonstrated that a strong signal was being sent to the cell to form autophagosomes. This agrees with our data showing that AR12 and neratinib interacted to cause autophagosome formation. The degradation and dephosphorylation of p70 S6K has important consequences for a cell over-expressing Tau and APP. Phosphorylation of ribosomal S6 is mediated by p70 S6K, and this enhances ribosomal protein synthesis. Inactivation of mTORC1 and p70 S6K, combined with enhanced phosphorylation of eIF2α S51 will likely completely shut down further synthesis of Tau and APP. In parallel, with enhanced autophagosome formation and autophagic flux, insoluble aggregates of Tau and APP will be cleared, restoring protein homeostasis to the neuron.

The co-chaperone BAG3 has been linked to the regulation of autophagy and cell viability, for example, Ji et al. demonstrated that BAG3 facilitates the autophagic degradation of Tau [30–32]. AR12 increased BAG3 expression and knock down of BAG3 reduced the ability of AR12 to cause autophagosome formation. Knock down of BAG3 profoundly reduced the ability of AR12 to reduce the expression of Tau and APP. BAG3 also facilitates the clearance of endogenous Tau in primary neurons and it plays a key role in sensing and regulating protein quality control [65]. Our data demonstrated that knock down of PERK / eIF2α significantly reduced the ability of AR12 to increase BAG3 expression. Furthermore, eIF2α is required to increase the expression of Beclin1 and ATG5 and knock down of eIF2α significantly reduces AR12-induced autophagosome formation. Knock down of Beclin1 / ATG5 / ULK1 also significantly reduced the AR12-induced expression of BAG3. These findings collectively support the hypothesis that AR12 via an initial catalytic inhibition of GRP78 results in an initial wave of ER stress signaling which leads to autophagosome formation. This results in a feed-forward positive loop where GRP78 and other chaperones are digested via autophagy resulting in greater levels of ER stress signaling, a significant increase in BAG3 expression, leading to greater autophagosome formation and the digestion of APP and Tau. Studies beyond the scope of the present manuscript will be required to fully define the relationship between the actions of AR12 and the biology of BAG3.

Our initial hypothesis, stated in the Introduction, was oversimplistic. Regulation of GRP78 function by both agents influences the abilities of the drugs to degrade APP and especially Tau. The relevance of BAG3 to the processes of degrading chaperones, APP and Tau was not initially appreciated, and our data highlight the importance of this cochaperone protein. Our prior publication and the data presented in this manuscript strongly suggest that AR12 and neratinib can cause the degradation of Tau and APP in multiple cell types, including microglia and neurons. Future work, based on the availability of funding, will be required to perform *in vivo* studies in transgenic Tau and APP mice to define whether these drugs have therapeutic efficacy against Alzheimer’s Disease mouse models. Should such studies eventually take place, we hope that this therapeutic combination can be tested for safety and activity in AD patients.

## Supplementary Material

Supplementary Figures
