# Supplementary material for: AR12 increases BAG3 expression which is essential for Tau and APP degradation via LC3-associated phagocytosis and macroautophagy
Source: Aging (Albany NY). 2022 Oct 13;14(20):8221–42. doi: 10.18632/aging.204337 (PMC9648812; doi:10.18632/aging.204337)
Supplement: Supplementary Figures [file aging-14-204337-s001.pdf]

SUPPLEMENTARY FIGURES

RAW Mϕ

	GRP78 (total)	GRP78 (surface)	HSP70	HSP90	HSP27	eIF2α (total)	eIF2α (P-S51)	ERK2
**+/+**								
VEH	100	100	100	100	100	100	100	100
AR12	77*	80*	87*	78*	87*	100	115#	100
NER	87*	84*	85*	87*	97	100	104	100
A+N	73*	73**	80*	65**	83*	100	127##	100
**-/-**								
VEH	100	100	100	100	100	100	100	100
AR12	95	99	96	98	97	97	102	100
NER	96	99	99	99	99	100	100	100
A+N	94	97	97	97	97	99	105	100

Supplementary Figure 1. Deletion of Rubicon prevents the degradation of chaperones, Tau and APP in macrophages. RAW macrophages (+/+ and -/- for Rubicon) were treated with vehicle control, AR12 (2 μM), neratinib (50 nM) or the drugs in combination for 6h. Cells were fixed in place and immunostaining performed to determine the expression of GRP78 (cell surface and total), HSP70, HSP90, HSP27, eIF2α and ERK2, and the phosphorylation of eIF2α S51. (n = 3 +/-SD) * $p < 0.05$ less than vehicle control; ** $p < 0.05$ less than corresponding neratinib value; # $p < 0.05$ greater than vehicle control; ## $p < 0.05$ greater than corresponding AR12 value.

A RAW Mϕ

	APP: CMV	APP: GRP78	APP: HSP70	APP: HSP90	ERK2: CMV	ERK2: GRP78	ERK2: HSP70	ERK2: HSP90
VEH	100	100	100	100	100	100	100	100
AR12	86*	92	90	87*	100	100	100	100
NER	89	100	94	91	100	100	100	100
A+N	74**	84*	84*	83*	100	100	100	99

	TAU: CMV	TAU: GRP78	TAU: HSP70	TAU: HSP90	ERK2: CMV	ERK2: GRP78	ERK2: HSP70	ERK2: HSP90
VEH	100	100	100	100	100	100	100	100
AR12	71*	87*†	85*†	84*†	100	100	100	100
NER	80*	92	87*	85*	100	99	100	100
A+N	68*	85*†	84*†	81*†	100	100	100	100

B RAW Mϕ

	APP: siSCR	APP: siGRP78	APP: siHSP70	APP: siHSP90	ERK2: siSCR	ERK2: siGRP78	ERK2: siHSP70	ERK2: siHSP90
VEH	100	100	100	100	100	100	100	100
AR12	88	76†	78*	81*	100	100	100	100
NER	98	89	92	88	100	100	100	100
A+N	77*	67†	73*	75*	100	100	100	100

	TAU: siSCR	TAU: siGRP78	TAU: siHSP70	TAU: siHSP90	ERK2: siSCR	ERK2: siGRP78	ERK2: siHSP70	ERK2: siHSP90
VEH	100	100	100	100	100	100	100	100
AR12	81*	67†	76*	73*	100	100	100	100
NER	87*	72†	76†	74†	100	100	99	99
A+N	71*	61†	70*	71*	100	100	99	99

Supplementary Figure 2. GRP78 plays a key role in regulating Tau and APP expression after drug exposure in macrophages. (**A**) RAW macrophages were transfected with an empty vector plasmid or with plasmids to express GRP78, HSP70 or HSP90, and in parallel co-transfected to express Tau or APP. After 24h, cells were treated with vehicle control, AR12 (2 μM), neratinib (50 nM) or the drugs in combination for 6h. Cells were fixed in place and immunostaining performed to determine the expression of Tau, APP and ERK2. (n = 3 +/-SD) * $p < 0.05$ less than vehicle control; ** $p < 0.05$ less than corresponding AR12 value; ¶ $p < 0.05$ greater than corresponding value in CMV transfected cells. (**B**) RAW macrophages were transfected to express APP or Tau and co-transfected with a scrambled siRNA or with an siRNA molecules to knock down the expression of GRP78, HSP70 or HSP90. After 24h, cells were treated with vehicle control, AR12 (2 μM), neratinib (50 nM) or the drugs in combination for 6h. Cells were fixed in place and immunostaining performed to determine the expression of Tau, APP and ERK2. (n = 3 +/-SD) * $p < 0.05$ less than vehicle control; † $p < 0.05$ less than corresponding value in siSCR cells.
